# Peroxiredoxin 6 Applied after Exposure Attenuates Damaging Effects of X-ray Radiation in 3T3 Mouse Fibroblasts

**DOI:** 10.3390/antiox10121951

**Published:** 2021-12-05

**Authors:** Elena G. Novoselova, Mars G. Sharapov, Sergey M. Lunin, Svetlana B. Parfenyuk, Maxim O. Khrenov, Elvira K. Mubarakshina, Anna A. Kuzekova, Tatyana V. Novoselova, Ruslan G. Goncharov, Olga V. Glushkova

**Affiliations:** Institute of Cell Biophysics of the Russian Academy of Sciences, 142290 Pushchino, Russia; sharapov.mg@yandex.ru (M.G.S.); lunin@rambler.ru (S.M.L.); lana_kras2@rambler.ru (S.B.P.); xpehob2004@mail.ru (M.O.K.); mubarakshina_e@rambler.ru (E.K.M.); 13krevetka@gmail.com (A.A.K.); novossulova_t@rambler.ru (T.V.N.); ruslangoncharov071@gmail.com (R.G.G.); glushckova@mail.ru (O.V.G.)

**Keywords:** X-ray radiation, 3T3 fibroblasts, proliferation, apoptosis, cellular stress, senescence, peroxiredoxin 6, Prdx6, radioprotector

## Abstract

Although many different classes of antioxidants have been evaluated as radioprotectors, none of them are in widespread clinical use because of their low efficiency. The goal of our study was to evaluate the potential of the antioxidant protein peroxiredoxin 6 (Prdx6) to increase the radioresistance of 3T3 fibroblasts when Prdx6 was applied after exposure to 6 Gy X-ray. In the present study, we analyzed the mRNA expression profiles of genes associated with proliferation, apoptosis, cellular stress, senescence, and the production of corresponding proteins from biological samples after exposure of 3T3 cells to X-ray radiation and application of Prdx6. Our results suggested that Prdx6 treatment normalized p53 and NF-κB/p65 expression, p21 levels, DNA repair-associated genes (XRCC4, XRCC5, H2AX, Apex1), TLR expression, cytokine production (TNF-α and IL-6), and apoptosis, as evidenced by decreased caspase 3 level in irradiated 3T3 cells. In addition, Prdx6 treatment reduced senescence, as evidenced by the decreased percentage of SA-β-Gal positive cells in cultured 3T3 fibroblasts. Importantly, the activity of the NRF2 gene, an important regulator of the antioxidant cellular machinery, was completely suppressed by irradiation but was restored by post-irradiation Prdx6 treatment. These data support the radioprotective therapeutic efficacy of Prdx6.

## 1. Introduction

It is well-known that ionizing radiation (IR) leads to the formation of free radicals and reactive oxygen species (ROS). Ionizing radiation induces cellular stress and damage mediated through either direct changes in DNA or indirect effects on DNA via generation of ROS [1]. Exposure of cells to IR may have various consequences, including cell death, mutations, transformation, and cell cycle arrest. Radiation-induced ROS cause single- and double-stranded DNA breaks and extensive base modifications. To evaluate cellular responses to IR, many different approaches have been used, ranging from chromosomal changes visualization to cell viability analysis, assessments of cell activity, and transcription profiling with an expression analysis of a large array of genes. Overall, this allows researchers to gain insight into the molecular mechanisms underlying the response to IR exposure.

The fate of irradiated cells is influenced by the activities of various transcription factors and interactions between them during the cell response to irradiation. Based on the fact that IR induces the active production of ROS in cells, it is reasonable to study effects of antioxidant enzymes, including peroxiredoxins, as radioprotectors. We have previously shown the beneficial effects of recombinant peroxiredoxin 6 (Prdx6, EC:1.11.1.27) in various pathologies associated with oxidative stress, such as mechanical and thermal skin injuries, chemical burns of the respiratory tract, ischemia-reperfusion injuries [2,3,4], and type 1 diabetes mellitus [5]. In the latter study, we demonstrated that Prdx6 protected RIN-m5F (rat insulinoma) beta cells cultured with high glucose levels through a mechanism that leads to a reduction in ROS production and apoptosis. It was shown that peroxiredoxins (Prdxs), an evolutionarily ancient family of peroxidases capable of reducing a wide range of inorganic and organic peroxide substrates, may play an important role in radioprotection [6,7]. It should be noted that we have recently demonstrated penetration of exogenous Prdx6 into the cells using FITC-labeled Prdx6 [8].

We also studied the radioprotective activity of Prdx6 in different models in vivo and in vitro, and these studies were associated with the prophylactic application of Prdx6 before exposure to IR [9,10,11,12]. In addition, we recently demonstrated that preliminarily applied Prdx6 protected 3T3 mouse fibroblasts against LD50 X-ray irradiation in vitro. Thus, pretreatment with Prdx6 increased cell survival, stimulated proliferation, normalized the level of ROS in the culture, and suppressed apoptosis and necrosis in 3T3 fibroblasts [8]. We believe that it is equally important to test whether Prdx6 is capable of exerting a radioprotective effect when applied several hours after irradiation. 

The effects of IR are the result of the activation of complex signaling pathway networks in response to DNA damage, which may lead either to recovery that is DNA repair and cell cycle arrest or cell death. These pathways are triggered by the activation of transcription factors, such as p53, nuclear erythroid-derived 2-related factor 2 (Nrf2), nuclear factor kappa B (NF-κB), and activating protein 1 (AP-1). Different radiation doses [13,14] and types of radiation produce different effects on gene expression [15]. High radiation doses are associated with increased severity of DNA damage, accompanied by responses to genotoxic stress, including the recognition of DNA damage, as well as altered repair mechanisms and immunological changes [16].

Among the transcription factors, nuclear factor kappa B (NF-κB) has been recognized as a key agent for the protection of cells against apoptosis in most cell types [17]. Both p53 and NF-κB are activated after exposure to IR, whereas activating protein 1 (AP-1) may control proliferation, aging, differentiation, and apoptosis, and Nrf2 may stimulate cellular antioxidant defense systems [18]. In addition, p21 was originally identified as a common inhibitor of cyclin-dependent kinases, transcriptionally modulated by p53, as well as a marker of cellular senescence. Earlier, p21 was considered a tumor suppressor that acted mainly by arresting the cell cycle and leading to the suppression of tumor growth. However, detailed studies of p21 have shown that p21 regulates responses to many cellular processes, including cell cycle arrest, apoptosis, DNA repair, aging, and autophagy [19].

In contrast, NF-κB has been shown to induce the activation of inflammatory and oxidative mediators, thus causing increased oxidative stress in cells [20,21]. Additionally, the transcription factor Nrf2, via the regulation of many antioxidant enzymes, such as glutathione peroxidase, may protect cells and tissues against inflammatory damage, mainly by inhibiting NF-κB signaling and suppressing the expression of several inflammatory and oxidative mediators [22,23].

Thus, the aim of this work was to study the effects of Prdx6 added to 3T3 fibroblasts several hours after irradiation of cells at a dose of 6 Gy. Thereby, the main subject of the work was not to identify the preventive effect of Prdx6, which has been demonstrated, but to investigate the possibility of its therapeutic activity when the enzyme is applied after irradiation. For this purpose, we analyzed mRNA expression profiles for genes associated with proliferation, apoptosis, senescence, and the production of corresponding proteins from biological samples after exposure to high doses of X-ray radiation and application of Prdx6. Finally, the aim of this study was to assess the role of Prdx6 in the regulation of therapeutic targets, such as NF-κB, Nrf2, TLR, p53, and p21, in irradiated fibroblasts to elucidate the therapeutic value of Prdx6 in counteracting X-ray toxicity. In parallel, we studied the responses of 3T3 cells by determining the production of cytokines IL-6 and TNF-α, expression of toll-like receptors (TLR1, TLR2, and TLR4), as well as the JNK pathway and SA-β-Gal activity. In addition, the stress response of the 3T3 cells was assessed using the heat shock protein system, including Hsp70, Hsp90α, and Hsp90β.

## 2. Materials and Methods

### 2.1. Cell Culture and Evaluation of Cell Proliferation

Cells of BALB/3T3 lineage (American Type Culture Collection) were seeded into 25 cm^2^ culture flasks (volume 5 mL), at concentration 1 × 10^6^ cells/flask, in DMEM (PanEco, Moscow, Russia) with addition of 10% fetal calf serum (Thermo, Swindon, UK) and an antibiotic/antimycotic solution (Sigma, Ronkonkoma, NY, USA). Cells were cultivated in a CO_2_ incubator at 37 °C and 5% CO_2_. For the experiments, cells of 5th–8th passages were used. Cells were allowed to attach for 24 h and then exposed to X-ray irradiation or a sham-irradiation (the same manipulations, excluding the X-ray device activation). Then, four hours later, 0.15 mg/mL Prdx6 was added.

Four groups of cells were used: (1) “control”, sham-irradiated 3T3 cells; (2) “Prdx6”, sham-irradiated 3T3 cells incubated in presence of Prdx6; (3) “6 Gy”, 3T3 cells irradiated with X-ray in dosage 6 Gy; (4) “6 Gy + Prdx6”, 3T3 cells irradiated with X-ray in dosage 6 Gy incubated in presence of Prdx6.

To assess survival, cells of 4 groups were placed into 96-well plates at concentration of 1 × 10^4^ cells/well and maintained at 37 °C and 5% CO_2_ for 24, 48, 72, or 120 h for subsequent survival evaluation, which included staining the cells with 0.05% Crystall Violet and counting using a Crystal Viollet counting (measure OD 595 nm) [5]. 

### 2.2. X-ray Treatment 

Irradiation was performed using a RUT-15 therapeutic X-ray device (focal length 8.5 cm, current 20 mA, voltage 200 kV) (Mosrentgen, Moscow, Russia) at a dose rate of 1 Gy/min. 3T3 cells were irradiated in culture flask, or 24, or 96-well plates at ambient temperature, and the accumulated dose was 6Gy. Sham-exposed cells were kept in the same conditions, excluding X-ray irradiation.

### 2.3. Isolation and Purification of the PRDX6

Genetic constructions encoding human Prdx6 enzymes were expressed in E. coli, strain BL21 (DE3), as described earlier [24]. The obtained recombinant proteins included a His-tag. The proteins were purified by affinity chromatography on Ni-NTA-agarose (Thermo Fisher Scientific, Waltham, MA, USA), as described in the manufacturer’s instructions. Isolation of proteins was performed as previously described [10]. The purity of the enzymes as measured by electrophoresis in 12% SDS/PAGE was at least 98%. Prdx6 diluted in phosphate buffer (1.7 mmol/L KH_2_PO_4_, 5.2 mmol/L Na_2_HPO_4_, 150 mmol/L NaCl, pH 7.4) at a concentration of 10 mg/mL was stored at −20 °C. Two months storage time at the above conditions produced no reduction in enzymatic activity. A peroxidase activity of Prdx6 in relation to hydrogen peroxide (H_2_O_2_) or tert-butyl hydroperoxide (t-BOOH) was according to Kang, with minor modifications. The peroxidase activity of recombinant Prdx6 was 230 nmol/min/mg of protein (in relation to H_2_O_2_) and 100 nmol/min/mg of protein (in relation to t-BOOH).

### 2.4. Senescence-Associated Beta-Galactosidase Staining

Cellular senescence of 3T3 cells exposed to X-ray radiation was detected using a senescence-associated β-galactosidase (SA-β-gal) assay. 3T3 cells cultured in 24-well plates at 1 × 10^4^ cells/well at 37 °C and 5% CO_2_ were treated as previously described [25]. After 120 h, the cells were washed with PBS and fixed in 2% formaldehyde/0.2% glutaraldehyde solution. The fixed cells were maintained overnight at 37 °C (without CO_2_) with SA-β-Gal staining solution. Finally, green blue–colored cells were counted (at least 100-200 cells per microscopic field in six fields) as a percentage of the total cell number and displayed as a percentage of cell senescence. 

### 2.5. Electrophoresis and Immunoblotting

3T3 cells were seeded into cell culture flasks (T25) at concentration of 1 × 10^6^ cells/flask, allowed to attach for 24 h and then exposed to X-ray irradiation or sham-irradiation. After irradiation (sham exposure) cells maintained at 37 °C and 5% CO_2_ for 120 h. Proteins from 3T3 cells were isolated using a lysis buffer as described previously [26]. The total protein concentration was measured using a spectrophotometer NanoDrop2000c (ThermoFisher Scientific, Wilmington, DE, USA). Equal quantities of total protein from samples were applied onto 10% SDS-PAGE and separated by electrophoresis. Then, a semi-dry transfer onto PVDF Hybond-P membranes (Amersham, Buckinghamshire, UK) was performed. Afterwards, the membranes were blocked using 5% fat milk in Tris-HCl buffer (pH 7.4) with 0.05% Tween 20 for 1 h, and monoclonal primary antibodies (1:1000) were applied, followed by incubation overnight at 4 °C. Following three washes with Tris-buffered saline/Tween 20, the membranes were maintained with an HRP-conjugated secondary goat anti-rabbit IgG antibody (P-GAR Iss, IMTEK, Moscow, Russia) (1:1000) for 1 h at ambient conditions. Primary monoclonal rabbit antibodies against GAPDH (14C10, #2118), NF-κB p65(C22B4, #4764), Phospho-NF-kB p65 Ser536 (93H1, #3033), Phospho-NF-kB p65 (Ser276) (93H1, #3037), Phospho-SAPK/JNK (Thr183/Tyr185 (#9251), Phospho-p53 (Ser46, #2521), Phospho-p53 (Ser15, #9284), p21 (#64016), Phospho-H2AX Ser139 (#2577), HSP70 (#4872), HSP90α (D1A7, #8165), HSP90β (#5087), Caspase-3 (#9662) (Cell Signaling Technology, USA) were used. GAPDH was used as a loading control. To develop blots, ECL Plus chemiluminescent cocktail (Amersham/GE) was used according to the manufacturer’s instructions. The blots were photographed using WL transilluminator (Vilber Lourmat, Collégien, France). Quantification of the protein bands was performed densitometrically with Image Studio Software ver. 5.2.5 (Li-COR, USA). The averaged results normalized to the corresponding loading control (GAPDH) were expressed in relative units. 

### 2.6. Gene Expression Analysis

3T3 cells were seeded into cell culture flasks (T25) at concentration of 1 × 10^6^ cells/flask, allowed to attach for 24 h, and then exposed to X-ray irradiation or sham-irradiation. After irradiation (sham exposure) cells maintained at 37 °C and 5% CO_2_ for 120 h. The gene expression level was determined by reverse transcription real-time PCR. Total RNA was isolated from 3T3 cells with ExtractRNA reagent (Evrogen, Moscow, Russia). RNA quality was estimated electrophoretically in 1.5% agarose gel. RNA concentration was determined using NanoDrop 1000c spectrophotometer (Thermo Fisher Scientific, Waltham, MA, USA). Two micrograms of total RNA were used for reverse transcription with MMLV reverse transcriptase and standard dT15 oligonucleotide (“Evrogen”, Russia). The synthesized cDNA was used for real-time PCR with qPCRmix-HS SYBR kit (“Evrogen”, Russia) and 200 nM gene-specific primers (Table S1). The genes expression related to the cellular antioxidant response system (SOD3, PRDX1, PRDX2, PRDX3, PRDX4, PRDX5, PRDX6), apoptosis (CASP3, p53), DNA repair system (APEX1, XRCC4, XRCC5, and Ogg1), senescence marker (CDKN1), some transcription factors (p65/NF-κB, NRF2, AP-1), Toll-like receptors (TLR1, TLR2, TLR4), heat shock proteins (HSP90 and HSP70), and cytokine IL-6 were analyzed. Real-time PCR was carried out using DNA amplifier DTlite (DNA-Technology, Moscow, Russia) with cycling mode: (1) «hot-start»: 95 °C, 5 min; (2) denaturation, 95 °C, 15 s; (3) primer annealing and DNA synthesis at 60°C, 30 s. Stages (2–3) were repeated 40 times. The expression levels of genes studied was normalized to that of the housekeeping gene-β-actin (ACTB). The 2^−∆∆Ct^ method was used to calculate differences in genes expression [27].

### 2.7. Measurement of Cytokine Production

3T3 cells of all groups were cultured in 24-well plates at 2 × 10^5^ cells/well at 37 °C and 5% CO_2_ for 120 h (for ELISA assay). The cytokine concentrations were determined in the cell lysates by ELISA method in 96-well plates. Commercial reagent kits for quantification of murine interleukin-6 (IL-6) or tumor necrosis factor (TNFα) were used (Peprotech, Rocky Hill, NJ, USA) as described earlier [28]. 

### 2.8. Statistical Analysis

Statistical data analysis was carried out using the Sigma Plot 11 software package (Systat Software Inc., San Jose, CA, USA). Statistical significance between experimental groups was determined using two-way ANOVA with Bonferroni post-hoc tests for survival analysis or unpaired Student’s *t*-tests for all other analyses. *p* < 0.05 was considered statistically significant. The results are presented as mean value ± standard error (SE).

## 3. Results

### 3.1. Effects of Prdx6 on the Survival, Proliferation, and Antioxidant Status of Irradiated 3T3 Cells

**Figure 1:**
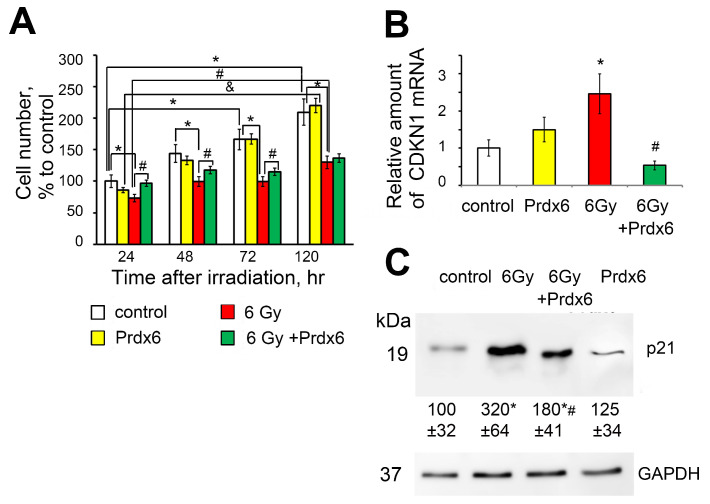
Effects of X-ray irradiation and Prdx6 addition on (**A**) cell proliferation (F_time_(9.105) = 18.569, *p* < 0.001; F_exposure_(3.105) = 240.29, *p* < 0.001; F_time__∗exposure_(3.105) = 133.92, *p* < 0.001); (**B**) mRNA level of CDKN1 in the 3T3 cells; (**C**) p21 protein level in 3T3 cells measured by Western blot analysis. Equal amounts of total proteins were analyzed with the corresponding antibodies with normalization to a GAPDH loading control (bottom). Blot images show a single representative experiment, while values below the protein bands show protein level in relative units corresponding to the internal GAPDH control calculated from 3 independent experiments, and all mRNA evaluation experiments were performed in 6 repetitions. * Significantly different from the sham-irradiated control, *p* < 0.05, & significantly different from the irradiated cells, *p* < 0.05, # significantly different from the irradiated cells, *p* < 0.05.

Prdx6 added to the 3T3 cell culture in vitro 4 h after irradiation of the cells with a dose of 6 Gy significantly reduced the radiation-induced cell’s death. A significant increase in cell survival was especially pronounced in the first two days after exposure to X-ray radiation in the group with the application of exogenous Prdx6 (4 h after irradiation). The number of viable cells in the Prdx6-treated irradiated group was 15–20% higher than in the irradiated control group (Figure 1A). The cyclin-dependent kinase inhibitor 1 (mRNA-CDKN1, protein-p21) was also increased after irradiation, while the presence of Prdx6 almost completely abolished this effect. At the same time, the application of exogenous Prdx6 to the culture of unirradiated 3T3 cells did not significantly affect the level of p21 expression (Figure 1B,C).

**Figure 2:**
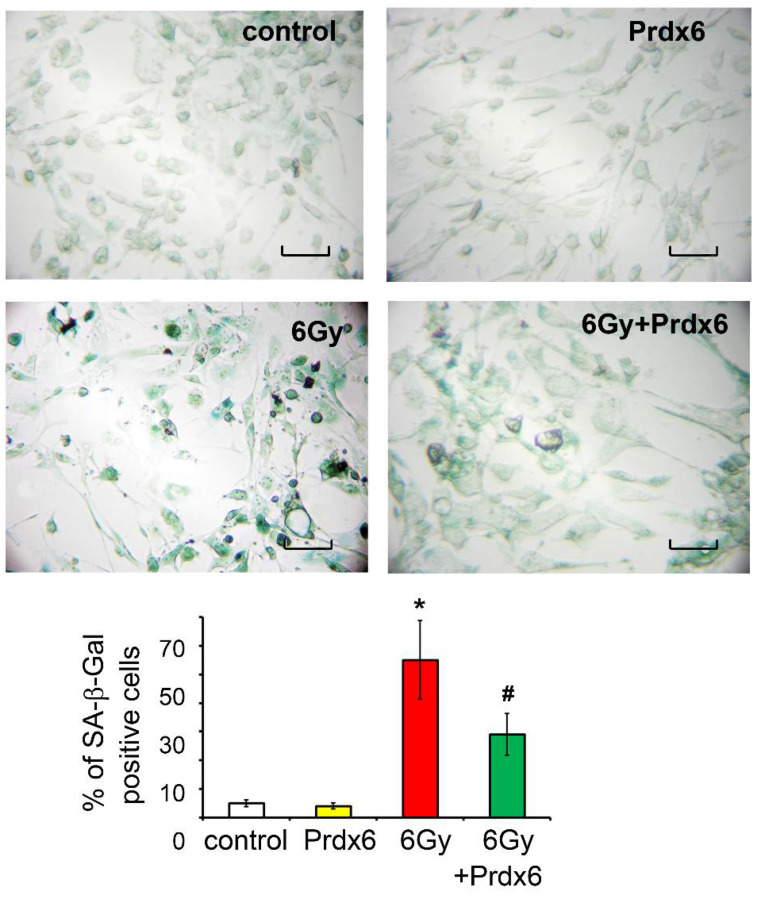
Representative images of effects of X-ray irradiation and Prdx6on SA-β-gal staining and the quantification of SA-β-gal-positive 3T3 cells. Data are percentages of total amounts of cells counted in the microscope’s field of vision, *n* = 300 or more ± SEM. Scale bars are 100 μm. * Significantly different from the sham-irradiated control, *p* < 0.05, # significantly different from the irradiated cells, *p* < 0.05.

Thus, the addition of Prdx6 prevented an increase of the senescence marker in irradiated 3T3 cells. In addition, evidence was obtained that the X-ray irradiation increased the percentage of SA-β-Gal positive cells, confirming the post-radiation oxidative stress and the activation of cell senescence mechanisms (Figure 2). In contrast, the addition of Prdx6 to the culture medium of the irradiated 3T3 cells led to a relative decrease in the percentage of SA-β-Gal-positive cells in comparison with non-treated irradiated cells. Meanwhile, the introduction of Prdx6 into the culture of non-irradiated cells did not affect the number of SA-β-Gal positive cells (Figure 2). 

**Figure 3:**
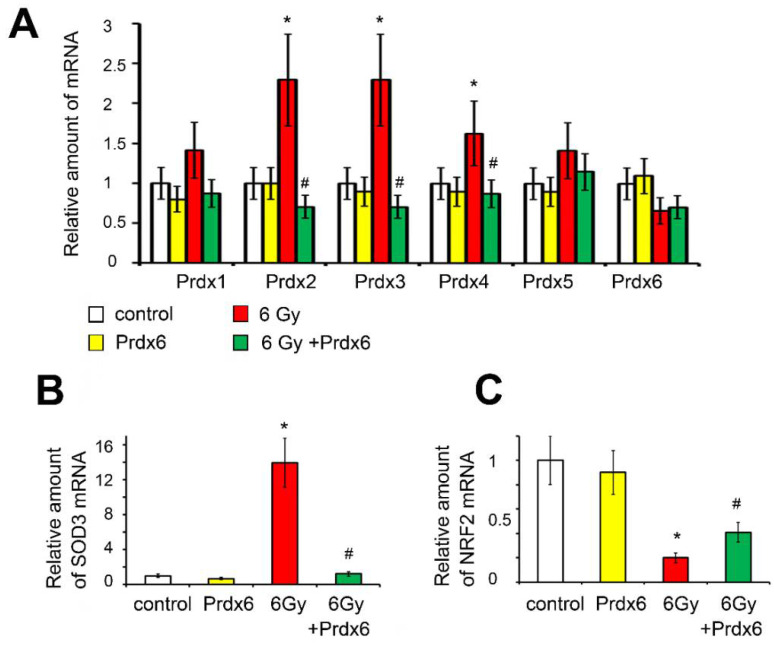
Effects of X-ray irradiation and Prdx6 addition on expression of genes regulating antioxidant status in 3T3 cells: (**A**) Peroxiredoxins; (**B**) SOD3; (**C**) NRF2. The mRNA evaluation experiments were performed in 6 repetitions. * Significantly different from the sham-irradiated control, *p* < 0.05, # significantly different from the irradiated cells, *p* < 0.05.

The expression of some antioxidant response system genes, for which the most significant change in expression after irradiation of 3T3 cells was previously shown, was assessed [8]. Interestingly, X-ray irradiation with a dose of 6 Gy resulted in a prolonged activation of the expression of isoforms PRDX2, PRDX3, and PRDX4, while the expression level of PRDX1, PRDX5, and PRDX6 did not differ significantly from the control values (Figure 3A). The addition of Prdx6 to the culture medium of 3T3 cells significantly normalized the expression of endogenous peroxiredoxins. The evaluation of SOD3 gene expression showed significant post-irradiation stimulation of the expression of this gene, whereas, in the presence of Prdx6, the expression of SOD3 in irradiated cells was almost normalized (Figure 3B). Opposite effects of radiation and Prdx6 were found for the expression of the NRF2 gene (Figure 3C).

### 3.2. Post-Irradiation Effects of Prdx6 on Cytokine Production, TLR’s Expression, Apoptosis, and Cellular Stress in Irradiated 3T3 Cells

**Figure 4:**
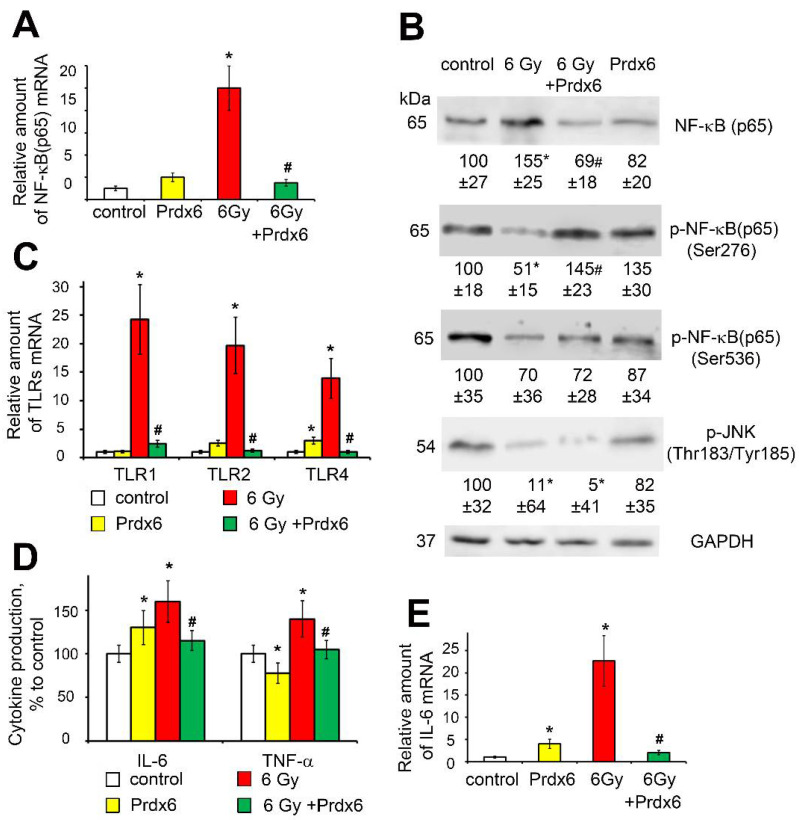
Effects of X-ray irradiation and Prdx6 addition on expression of genes and levels of proteins regulating and characterizing the immune status in the 3T3 cells: (**A**) NF-κB mRNA level; (**B**) NF-κB, p-NF-κB (Ser276 and Ser536, were normalized with total p65) and JNK proteins level; (**C**) TLRs mRNA level; (**D**) IL-6 mRNA level and TNF-α and IL-6 production in the 3T3 cells; (**E**) mRNA IL-6 level. The explanations for Western blot and mRNA analysis as in the legend for Figure 1. * Significantly different from the sham-irradiated control, *p* < 0.05, # significantly different from the irradiated cells, *p* < 0.05.

By evaluating the effects of radiation on the 3T3 cell’s activity, we observed that X-ray radiation with a dose of 6 Gy led to the activation of the NF-κB pathway, increased the production of pro-inflammatory cytokines IL-6 and TNF-α, and increased the expression of toll-like receptors TLR1, TLR2, and TLR4 (Figure 4). Notably, the administration of Prdx6 after irradiation removed the pro-inflammatory effects of radiation, both at the gene and protein levels. The only exception was a JNK signaling cascade, whose activity did not increase, but decreased by almost 10 times after irradiation, while the addition of Prdx6 did not change the effect of radiation on JNK activity (Figure 4B).

**Figure 5:**
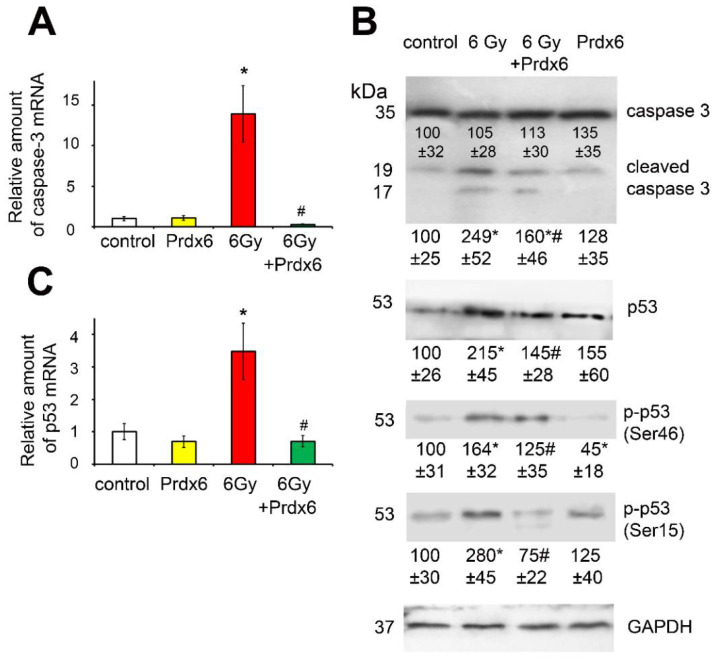
Effects of X-ray irradiation and Prdx6 addition on expression of genes and levels of proteins regulating and characterizing apoptosis in 3T3 cells: (**A**) mRNA of caspase 3; (**B**) p53, p-p53 (was normalized with total p53), and caspase 3 protein level; (**C**) mRNA of p53. The explanations for Western blot and mRNA analysis as in the legend for Figure 1. * Significantly different from the sham control cells, *p* < 0.05, # significantly different from the irradiated cells, *p* < 0.05.

Apoptosis in irradiated 3T3 fibroblasts was assessed by the expression and activity of caspase 3 (Figure 5A,B). The results showed that IR sharply accelerated apoptosis in 3T3 cells, and the addition of Prdx6 to the cell culture medium decreased the expression of the gene regulating the production of caspase 3 below the control level, which indicated the ability of Prdx6 to protect cells from radiation-induced apoptosis. Additionally, the production of the p53 protein was assessed in 3T3 cells, as well as levels of phosphorylated forms of this protein, ph-p53 (S46) and ph-p53 (S15), which have different roles in the cell. It was shown that IR significantly increased the total level of p53 in the cells, as well as the phosphorylation of p53 at Ser 46 and Ser 15 (Figure 5B,C). In addition, the Prdx6 added to the cells 4 h after irradiation demonstrated an obvious protective effect, manifesting in the normalization of the p53 level, as well as in a tendency to restoring of ph-p53 (S46) and ph-p53 (S15) levels, especially the ph-p53 (S15) form, which promotes cell survival [29].

**Figure 6:**
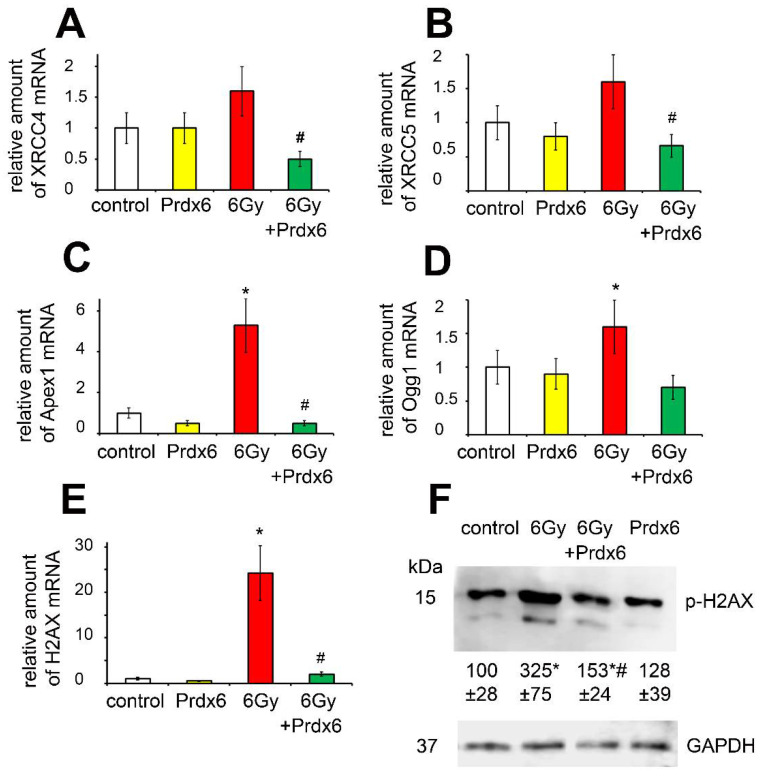
Effects of X-ray irradiation and Prdx6 addition on expression of genes and level of proteins regulating and characterizing DNA reparation in the 3T3 cells: (**A**) mRNA of XRCC4; (**B**) mRNA of XRCC5; (**C**) mRNA of Apex 1; (**D**) mRNA of Apex 1; (**E**) mRNA of H2AX; (**F**) The level of p-H2AX protein. The explanations for Western blot and mRNA analysis as in the legend for Figure 1. * Significantly different from the sham-irradiated control, *p* < 0.05, # significantly different from the irradiated cells, *p* < 0.05.

We also evaluated the expression of a panel of DNA repair-associated genes (such as XRCC4, XRCC5, Apex1, and Ogg1) (Figure 6). The results showed that IR stimulates the expression of these genes, indicating DNA damage in the 3T3-irradiated cells. In addition, irradiation led to increase in phosphorylation of H2AX which is a clear indication of DNA damage. Along with that, IR modulated the expression of H2AX that may indicate its effects on global histone regulation. Prdx6 protein added to the culture medium after irradiation exerted a protective effect in cells, which was indicated by a decrease in the expression of these genes associated with DNA damage and cell senescence, as well as a decrease in p-H2AX level, an important marker of IR-induced double-strand DNA breaks [30,31].

**Figure 7:**
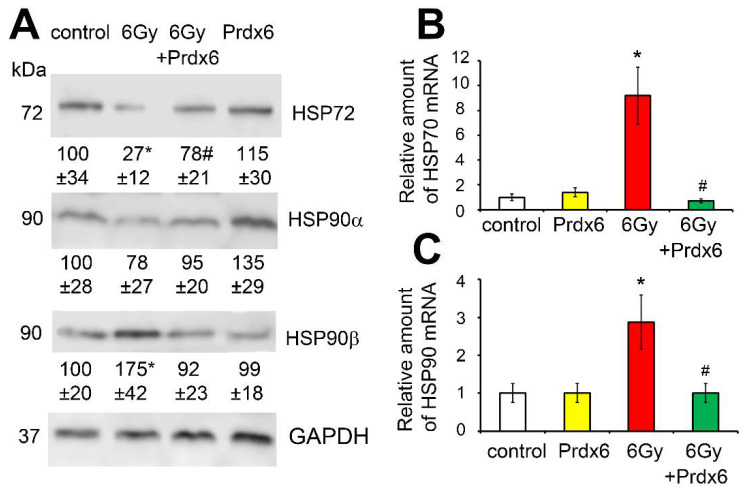
Effects of X-ray irradiation and Prdx6 addition on expression of genes and levels of proteins, regulating and characterizing heat shock proteins in the 3T3 cells: (**A**) HSP90α and HSP90β proteins levels; (**B**) mRNA of HSP70; (**C**) mRNA of HSP90. The explanations for Western blot and mRNA analysis as in the legend for Figure 1. * Significantly different from the sham-irradiated cells, *p* < 0.05, # significantly different from the irradiated cells, *p* < 0.05.

The expression of genes regulating the production of heat shock proteins HSP90α, HSP90β, and HSP70 is a direct indicator of cellular stress. We found that the irradiation of 3T3 fibroblasts led to the significant activation of genes that regulate the production of the inducible form of the heat shock proteins HSP90α, HSP90β, and HSP70 (Figure 7). The presence of Prdx6 in the cell culture medium prevented the stress response of 3T3 cells to X-ray irradiation.

## 4. Discussion

Earlier, we demonstrated that preliminary administration of exogenous Prdx6 before irradiation of cells [8] and animals [10] provided a radioprotective effect. The radioprotective effect of Prdx6 approximately for 80% was due to its peroxidase activity, and, for 20%, due to stimulation of the TLR4 receptor [8]. It should be noted that exposure to ionizing radiation produces a long-term oxidative stress. In particular, after irradiation, a prolonged (hours-days) increase in the level of lipid peroxidation was observed [32], as well as formation of long-lived reactive protein species [33] that were shown to be effectively eliminated by Prdx6 [10]. In addition, after exposure to radiation, a dysfunction of the electron transport chain of mitochondria and the activation of a number of oxidases (NAD (P) H-oxidase, xanthine oxidase, cyclooxygenase etc.) were observed, which also contribute to an increase in the level of intracellular ROS and the progression of oxidative stress [34]. In this regard, it was interesting to test the effects of antioxidant enzyme Prdx6 applied after exposure to X-rays. Therefore, the purpose of this study was to evaluate the radiomitigating properties of recombinant Prdx6 in the culture of 3T3 embryonic fibroblasts. 

Prdx6 can neutralize the broadest range of hydroperoxides and, unlike other members of the Prdx family, is able to reduce phospholipid peroxides and peroxynitrite, as well as phospholipase A2 activity (aiPLA2) under certain conditions [35]. Apparently, due to the aiPLA2 activity, Prdx6 may penetrate into cells, thereby directly affecting their redox status [8].

In cells that survive X-ray irradiation, changes in the expression of genes associated with DNA repair, cell cycle, inflammation, and immune responses are usually observed [36]. To study the effects of recombinant Prdx6 protein on irradiated 3T3 cells, we measured the key regulators of cellular processes. Among them, nuclear transcription factor kappa B (NF-κB) is a key factor in the regulation of metabolic pathways in most cell types. NF-κB is a central transcription factor in the immune system and influences cell survival. Moreover, the induction of radioresistance is mediated by several NF-κB regulated genes [17]. The p53 protein plays an important role in the regulation of the cell cycle, DNA repair, and apoptosis and is an attractive therapeutic target for cancer treatment [37]. Surprisingly, we have shown that both p53 and NF-κB are activated in 3T3 cells after exposure to IR, whereas the presence of Prdx6 significantly reduced the effect of X-ray irradiation.

When studying the expression of PRDX1-6, which are considered the most important intracellular hydroperoxidases, it was found that isoform-specific expression of peroxiredoxins was induced on 5th day after irradiation (Figure 3), which may be explained by adaptation to the changing spectrum of hydroperoxides in the cell, because peroxiredoxin isoforms have different efficacy towards different peroxide substrates. However, in general, the induction of the expression of these genes was several times lower than in the first 6 h after irradiation of 3T3 cells [8], which may be due to the suppression of NRF2 (Figure 3), which regulates the expression of genes of many antioxidants’ enzymes [38,39]. It should be noted that suppression of NRF2 may be mediated via activation of the transcription factor NF-κB (Figure 4), which is shown to suppress NRF2 activity [40]. The activation of NF-κB may explain the increase in the expression of some genes, which are controlled by NF-κB, related to the antioxidant response (SOD3) [41] and DNA repair (XRCC4, XRCC5, H2AX, Ogg1, and Apex1) [40].

The panel of DNA repair-associated genes in the irradiated 3T3 fibroblasts was markedly activated, whereas, after the Prdx6 addition, the activation of the gene panel was significantly reduced (Figure 6), which may indicate a decrease in ROS-induced oxidative DNA damage in the presence of Prdx6. These results support the radioprotective efficacy of Prdx6.

Moreover, the anti-inflammatory effect of Prdx6 in irradiated 3T3 fibroblasts was demonstrated. Indeed, while irradiation induced the expression of toll-like receptors (TLR1, TLR2, and TLR4), which is consistent to previous data [42], the production of pro-inflammatory cytokines IL-6 and TNFα, cell senescence (assessed by SA-β-Gal staining), and Prdx6 almost completely removed the pro-inflammatory effects of X-ray irradiation.

One of the important regulators of cellular activity is a low-molecular-weight p21 protein, transcribed from the CDKN1A gene, which was first described as a cyclin-dependent kinase 1 (CDKN1) inhibitor. It plays an important role in cell cycle control [43]. p21 stops cell cycle progression during G1 and S-phases via the binding and inhibition of cyclin-CDKN1,2,4,6 complexes [44]. Indeed, we have shown that X-ray irradiation at a dose of 6 Gy significantly increased CDKN1 gene expression, p21 production, and p21 phosphorylation, while the addition of Prdx6 into the culture medium of the X-ray irradiated fibroblasts normalized the proliferation of the 3T3 cells.

In addition, we have shown that genes regulating the production of heat shock proteins HSP90 and HSP70 were activated in the irradiated cells, with an inducible form of the HSP90 protein, HSP90β, with the most noticeable activation. Since these proteins are markers of cellular stress, we may conclude that IR induces cellular stress. It should be noted that Prdx6 also acts as a radioprotector, reducing the cellular stress caused by irradiation. 

**Figure 8:**
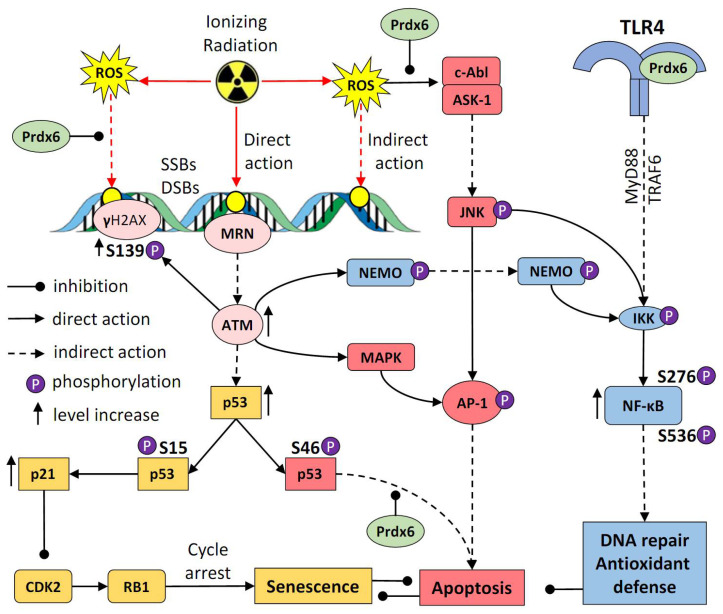
Schematic representation of the action of exogenous Prdx6 applied after exposure to X-rays. Ionizing radiation directly and indirectly (via ROS) causes single-stranded (SSBs) or double-stranded (DSBs) DNA breaks. The MRN complex (Mre11, Rad50, and Nbs1 proteins) recognizes DNA damage and activates ataxia telangiectasia-mutated (ATM) kinase. ATM phosphorylates histone H2AX at Ser139 (γH2AX), as well as checkpoint kinases CHK1 and CHK2, which phosphorylate p53. Phosphorylation of p53 at Ser15 leads to cell cycle arrest, while phosphorylation at Ser46 promotes apoptosis. In turn, p-p53 (ar Ser15) promotes the expression of p21 (cyclin-dependent kinase inhibitor p21 (CDKN1A)), which inhibits cyclin-dependent kinase 2 (CDK2), thereby inhibiting phosphorylation of retinoblastoma (Rb) protein and causing cell arrest. Exogenous Prdx6 prevents increase in the level of intracellular ROS by their elimination in the extracellular space, as well as directly inside the cell after Prdx6′s penetration into cytoplasm [8]. Thus, the recombinant Prdx6 inhibits an increase of DNA damage and p21 activation, preventing the development of senescence. Prdx6 also prevents apoptosis by suppressing ROS-mediated activation of the ASK-1/JNK/AP-1 signaling pathway and an increase in the level of p-p53 (Ser46). An important role in the suppression of apoptosis is played by NF-κB, which is activated with the participation of NEMO (NF-κB essential modulator), and stimulation of the TLR4 receptor by exogenous Prdx6.

Advantages of new radioprotector development are related not only to providing protection in “working spaces” or during incidents of radioactive contamination but also to the use of radiation therapy [45]. Radiation therapy is currently one of the main treatments for cancer; despite the many benefits of this treatment, such as non-invasiveness, preservation of organ integrity, and precision when targeting a tumor, it can lead to complications in the irradiated healthy tissue. Therefore, applying radioprotective means may alleviate radiation-induced complications. Although many studies have aimed to identify radioprotective agents [46], there is still a need for new effective radioprotectors. Previously, we demonstrated that the radioprotective effects of Prdx6 are based on its capability for ROS neutralization and, potentially, on its ability to activate signaling regulatory mechanisms for the restoration of unbalanced redox homeostasis [10]. The summary on the protective effects of exogenous Prdx6 in the irradiated cells is shown in Figure 8. This study additionally supports this conclusion, importantly, using the post-radiation administration of the recombinant antioxidant protein peroxiredoxin 6, which may be a promising radioprotector/ radiomitigator. 

## Data Availability

Data is contained within the article or Supplementary Materials.

## Supplementary Materials

The following are available online at https://www.mdpi.com/article/10.3390/antiox10121951/s1, Table S1: Oligonucleotides used for real-time PCR.
