# Supplementary material for: Peroxiredoxin 6 Applied after Exposure Attenuates Damaging Effects of X-ray Radiation in 3T3 Mouse Fibroblasts
Source: Antioxidants (Basel). 2021 Dec 5;10(12):1951. doi: 10.3390/antiox10121951 (PMC8750386; doi:10.3390/antiox10121951)
Supplement: Supplementary file 1 [file antioxidants-10-01951-s001.zip › antioxidants-1461414-supplementary.pdf]

Supplementary Materials

Table S1. Oligonucleotides used for real-time PCR.

Gene	GenBank	Primer Sequence	Localization
ACTB	NM_007393.4	F: CCTTCCTTCTTGGGTATGGAATCC<br>R: CACCAGACAGCACTGTGTTGGCA	899-922<br>1013-991
NRF2	NM_010902	F: CTCGCTGGAAAAAGAAGTG<br>R: CCGTCCAGGAGTTCAGAGG	4260-4278<br>4499-4482
NF-kB (p65)	NM_009045.5	F: CCAGACACAGATGATCGCCA<br>R: CGGGGTTCAGTTGGTCCATT	1162-1181<br>1268-1249
c-JUN (AP-1)	NM_010591	F: CACGGAGAAGAAGCTCACAA<br>R: ACTTGTTACCGGTCCTCTGG	240-259<br>365-346
CASP-3	NM_009810	F: AAGGAGCAGCTTTGTGTGTG<br>R: GAAGAGTTTCGGCTTTCCAG	556-575<br>700- 681
TP53	NM_011640.3	F: CGAAGACTGGATGACTGCCA<br>R: CGTCCATGCAGTGAGGTGAT	148-167<br>284-265
CDKN1A	NM_007669.5	F: GTCTTGCACTCTGGTGTCTGA<br>R: TAGAAATCTGTCAGGCTGGTCT	443-463<br>541- 520
IL-6	NM_031168	F: TAGTCCTTCCTACCCCAATTTCC<br>R: TTGGTCCTTAGCCACTCCTTC	566-588<br>641-621
TLR1	NM_030682.2	F: GCCAAACGCAAACCTTACCAG<br>R: AGCTTGGACAATGAGAGGATGT	270-290<br>389-368
TLR2	NM_011905.3	F: CTACATTGGCCATGGTGACCT<br>R: TGCCCAGAGAATAAAAGGCGT	547-567<br>653-633
TLR4	NM_021297.3	F: CCCTGCATAGAGGTAGTTCCTAA<br>R: CTTCAAGGGGTTGAAGCTCAGAT	360-382<br>479-457
XRCC4	NM_028012	F: GAGACACCGAATGCAGAAGA<br>R: GGTGCTCTCCTCTTTCAAGG	920-939<br>1040-1021
XRCC5	NM_009533	F: GAAGAACAGCGCTTCAACAG<br>R: TCCTGAACAACAATTTCCCA	2080-2099<br>2171-2152
Ogg1	NM_010957.4	F: AGATTGCCCATCGTGACTACG<br>R: TCCGGAAAAAGTTTCCCAGTTC	1068-1088<br>1163-1142
Apex1	NM_009687.2	F: GGTCAGCTCCGTCAGACAAA<br>R: TCCTCGCCAATGCCATAAGA	491-510<br>581-562
H2AX	NM_010436.2	F: TCAGGAGTACTGAGGGGGC<br>R: TGGCTCAGCTCTTTCTGTGAG	467-485<br>571- 551
HSP70	NM_024172	F: CACGTTCGACGTGTCCATCCTG<br>R: ACCAGCCGGTTGTCGAAGTCCT	615-636<br>719-698
HSP90	NM_011631	F: GTCCGCCGTGTGTTCATCAT<br>R: GCACTTCTTGACGATGTTCTTGC	1164-1183<br>1331-1309
SOD3	NM_011435	F: CTGAGGACTTCCCAGTGAC<br>R: GGTGAGGGTGTCAGAGTGT	1456-1473<br>1651-1634
PRDX1	NM_011034	F: AATGCAAAAATTGGGTATCCTGC<br>R: CGTGGGACACACAAAAGTAAAGT	86-108<br>235-213
PRDX2	NM_011563	F: CACCTGGCGTGGATCAATACC<br>R: GACCCCTGTAAGCAATGCCC	390-410<br>556-537

PRDX3	NM_007452	F: GGTTGCTCGTCATGCAAGTG<br>R: CCACAGTATGTCTGTCAAACAGG	107-126<br>206-184
PRDX4	NM_016764	F: CTCAAACTGACTGACTATCGTGG<br>R: CGATCCCCAAAAGCGATGATTTC	408-430<br>508-486
PRDX5	NM_012021	F: GGCTGTTCTAAGACCCACCTG<br>R: GGAGCCGAACCTTGCCTTC	772-792<br>925-907
PRDX6	NM_177256.5	F: TAAGGACAGGGACATTTCCATCC<br>R: CCGTGGAGTTAGGGTAGAGGA	694-716<br>839-819
